# Alleviation of carbon catabolite repression in *Enterobacter aerogenes* for efficient utilization of sugarcane molasses for 2,3-butanediol production

**DOI:** 10.1186/s13068-015-0290-3

**Published:** 2015-07-31

**Authors:** Moo-Young Jung, Hwi-Min Jung, Jinwon Lee, Min-Kyu Oh

**Affiliations:** Department of Chemical and Biological Engineering, Korea University, Seoul, Republic of Korea; Department of Chemical and Biomolecular Engineering, Sogang University, Seoul, Republic of Korea

**Keywords:** 2,3-Butanediol, *Enterobacter aerogenes*, Sugarcane molasses, Fed-batch fermentation, Catabolite repressor/activator, Carbon catabolite repression

## Abstract

**Background:**

Due to its cost-effectiveness and rich sugar composition, sugarcane molasses is considered to be a promising carbon source for biorefinery. However, the sugar mixture in sugarcane molasses is not consumed as efficiently as glucose in microbial fermentation due to complex interactions among their utilizing pathways, such as carbon catabolite repression (CCR). In this study, 2,3-butanediol-producing *Enterobacter aerogenes* was engineered to alleviate CCR and improve sugar utilization by modulating its carbon preference.

**Results:**

The gene encoding catabolite repressor/activator (Cra) was deleted in the genome of *E. aerogenes* to increase the fructose consumption rate. However, the deletion mutation repressed sucrose utilization, resulting in the accumulation of sucrose in the fermentation medium. Cra regulation on expression of the *scrAB* operon involved in sucrose catabolism was verified by reverse transcription and real-time PCR, and the efficiency of sucrose utilization was restored by disrupting the *scrR* gene and overexpressing the *scrAB* operon. In addition, overexpression of the *ptsG* gene involved in glucose utilization enhanced the glucose preference among mixed sugars, which relieved glucose accumulation in fed-batch fermentation. In fed-batch fermentation using sugarcane molasses, the maximum titer of 2,3-butanediol production by the mutant reached 140.0 g/L at 54 h, which was by far the highest titer of 2,3-butanediol with *E. aerogenes* achieved through genetic engineering.

**Conclusions:**

We have developed genetically engineered *E. aerogenes* as a 2,3-butanediol producer that efficiently utilizes sugarcane molasses. The fermentation efficiency was dramatically improved by the alleviation of CCR and modulation of carbon preference. These results offer a metabolic engineering approach for achieving highly efficient utilization of mixed sugars for the biorefinery industry.

**Electronic supplementary material:**

The online version of this article (doi:10.1186/s13068-015-0290-3) contains supplementary material, which is available to authorized users.

## Background

The development of biorefineries has attracted a great deal of interest due to increasing energy costs and environmental concerns resulting from fossil fuel utilization [1, 2]. As an example, microbial production of 2,3-butanediol has been intensively studied in the past few years due to its multiple industrial applications, including the production of synthetic rubber, plasticizers, fuel additives, and fumigants [3, 4]. In microbial fermentation for 2,3-butanediol production, the carbon source is one of the major drivers of cost. Therefore, much effort has been made to find inexpensive feedstocks, such as corncob [5], jatropha hulls [6], Jerusalem artichoke tubers [7], and molasses [8]. In general, most biomass derived from lignocellulose and waste materials contains a few mixed sugars. For example, the hydrolysis of lignocellulosic biomass produces a mixture of sugars containing glucose, xylose, and arabinose [9]. The main carbohydrate of Jerusalem artichoke tuber is inulin, which can be hydrolyzed by inulinase to fructose and glucose [10].

When provided with a mixture of different carbon sources, most microorganisms prefer to use one carbon source for the fastest growth. The presence of preferred carbon sources often prevents the utilization of secondary substrates by the regulatory mechanisms, including transcription activation or repression of certain genes concerned with the use of alternative carbon sources, which is called carbon catabolite repression (CCR) [11, 12]. In enteric bacteria, two dominant CCR mechanisms involve transcriptional regulation by cyclic AMP receptor protein (Crp) and by catabolite repressor/activator (Cra) [13]. Crp is known to regulate the genes involved in carbon metabolism, such as lactose, arabinose, mannose, glucosamine, and amino sugars operons, in response to the depletion of preferred carbon source [14, 15]. On the other hand, Cra generally regulates carbon flux through by repression of genes encoding glycolytic pathway enzymes, or by activating key genes involved in the Krebs cycle, glyoxylate shunt, gluconeogenic pathways and electron transfer [16]. After the preferred carbon source is depleted, an intermediate lag-phase is caused by a shift in the catabolic system to utilize a secondary sugar. Because the catabolic characteristic of microbial fermentation with mixed sugars results in a delayed and complicated fermentation process, alleviation of CCR of a host microorganism is very helpful for improving its fermentation efficiency [17].

Sugarcane molasses contains several mixed sugars, a dominant amount of sucrose, and similar amounts of glucose and fructose. It is considered to be a promising feedstock for biorefinery due to its rich sugar content and cost-effectiveness [18]. In our previous study, disruption of the *scrR* gene, a transcriptional repressor of the *scr* regulon for sucrose catabolism, was conducted in *Enterobacter aerogenes* for efficient utilization of sucrose in sugarcane molasses for 2,3-butanediol fermentation [19]. The *scrR* mutation increased the sucrose consumption rate significantly, resulting in 2,3-butanediol production from sugarcane molasses that was enhanced by 56.8% in batch fermentation over its parent strain. In addition, 98.7 g/L of 2,3-butanediol production was achieved at 36 h of fed-batch fermentation with molasses feeding. However, despite these advances, several obstacles remain for more efficient utilization of sugarcane molasses in fed-batch fermentation. First, the efficiency of fructose utilization in the *scrR* mutant was relatively reduced, which resulted in a significant amount (~30 g/L) of fructose accumulation in the medium after the fermentation period. Second, the fermentation duration of the *scrR* mutant with sugarcane molasses for 2,3-butanediol production was shorter by 33.3% than that with glucose, which might be caused by cell stress from the repeated catabolic shift between consumed sugars (glucose and sucrose), according to the feeding of sugarcane molasses.

**Fig. 1:**
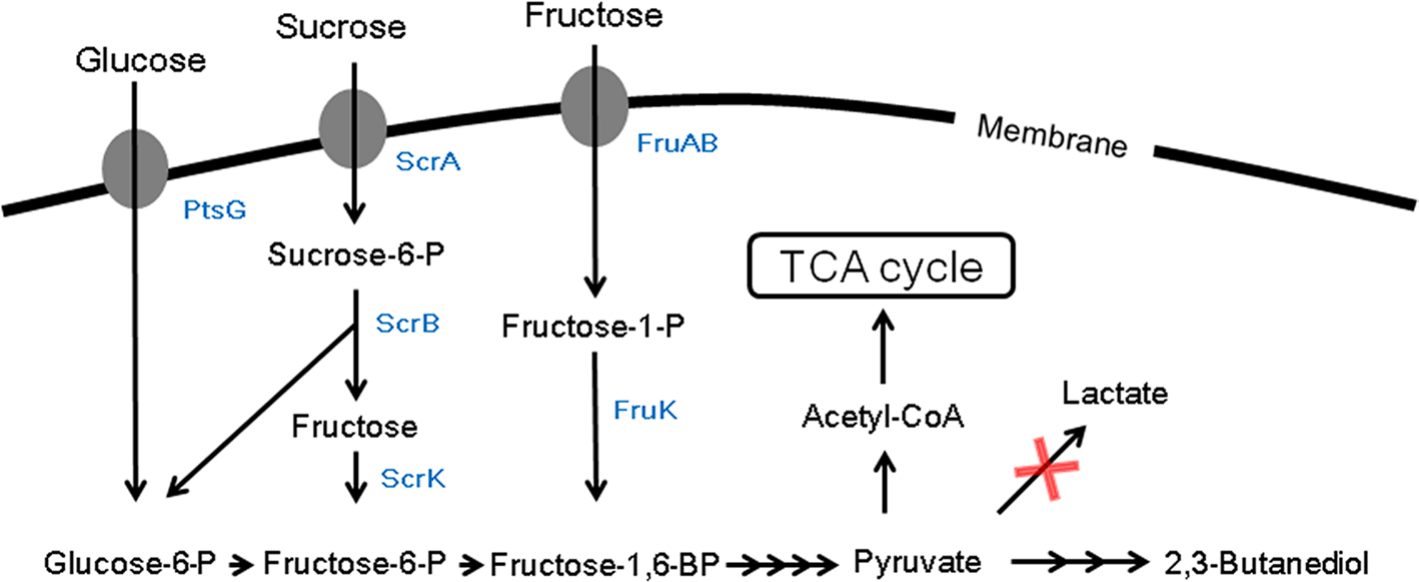
Catabolic pathway of PTS-mediated sugars contained in sugarcane molasses in *E. aerogenes. Symbols* represent deleted genes (*red cross)*. *ScrA* EII transport protein for sucrose, *ScrB* sucrose-6-phosphate hydrolase, *ScrK* fructokinase, *PtsG* EIIBC^Glc^ complex, *FruA* EIIBC^fru^ complex, *FruB* EIIA^fru^ component, *FruK* fructose-1-phosphate kinase.

Therefore, the purpose of this study was to develop a 2,3-butanediol-producing mutant that can utilize all three sugars contained in sugarcane molasses efficiently and simultaneously. As shown in Fig. 1, all sugars in molasses (glucose, fructose, and sucrose) are transported into cells by the phosphotransferase system (PTS) and then converted to intermediates, such as glucose-6-phosphate (G6P), fructose-6-phosphate (F6P), and fructose-1,6-bisphosphate (F1,6BP), involved in the preparatory phase of glycolysis [20–23]. Transcription of the *fruBKA* operon, encoding genes for fructose uptake and utilization, was negatively regulated by Cra, previously designated as *fruR* [24]. When glucose is depleted, Cra activates the metabolic pathways utilizing gluconeogenic carbon sources, such as acetate, pyruvate, glycerol, and glucogenic amino acids [25, 26].

In this study, deletion of the *cra* gene in *E. aerogenes* showed a much higher fructose utilization rate, but significantly retarded sucrose utilization. The reduced sucrose consumption in *cra*-deficient *E. aerogenes* was examined by reverse transcription real-time PCR. In addition, the carbon preference and efficiency were modulated by overexpression of the key genes involved in each carbon catabolism, which reduced the accumulation of certain carbon sources in fed-batch fermentation. The catabolic regulation of carbohydrates involved in sugarcane molasses and the strategies for constructing 2,3-butanediol production strain are shown in the Additional file 1.

## Results and discussion

### Effects of *cra* deletion on sugar utilization and metabolite production

To improve fructose utilization, disruption of *cra* was performed. An open-reading frame that contained a 95% amino acid sequence identity to Cra of *Escherichia coli* K-12 MG1655 was identified from the *E. aerogenes* genome using BLAST (Basic Local Alignment Search Tool). The deletion of *cra* was performed from EMY-01 (*△ldhA*) and EMY-68 (*△ldhA △scrR*), respectively, and was verified by colony PCR (data not shown). The resulting strains were named EMY-69 (*△ldhA △cra*) and EMY-70 (*△ldhA △scrR △cra*), respectively (Table 1). In our previous study, the *ldhA*-deficient *E. aerogenes* mutant (EMY-01) showed improved carbon flux and NADH availability for 2,3-butanediol production [27]. In addition, acidification of the culture medium was alleviated due to reduced lactate production. Because of these advantages, EMY-01 was used as a control strain in this study.

**Table 1:** Strains and plasmids used in this study

Strains or plasmids	Genotype or relevant characteristics	Source or reference
Strain		
*E. coli* DH5α		Invitrogen
*E. aerogenes* KCTC 2190	Wild type	Korean Collection for Type Culture
EMY-01	*E. aerogenes* KCTC 2190 *△ldhA*	[27]
EMY-68	*E. aerogenes KCTC 2190 △ldhA △scrR*	[19]
EMY-69	*E. aerogenes KCTC 2190 △ldhA △cra*	This study
EMY-70	*E. aerogenes KCTC 2190 △ldhA △scrR △cra*	This study
EMY-70S	Plasmid-based *scrAB* overexpression strain of EMY-70 by introduction of pZS21MCS::*scrAB*	This study
EMY-70SP	Plasmid-based *ptsG* overexpression strain of EMY-70S by introduction of pZA31MCS::*ptsG*	This study
Plasmid		
pKM208	lacI, *λ* Red + Gam-producing vector, tac_promoter, f1_ori, *Amp* ^R^	Addgene
pCP20	FLP recombinase-producing vector, cI857, pSC101 ori, *Amp* ^R^, *Cm* ^R^	[40]
pKD4	FRT flanked resistance cassette involved vector, oriRγ, *Km* ^R^	[40]
pZA31MCS	*E. coli*—*K. pneumoniae* shuttle vector, P_LtetO-1_, p15A ori, Cm^R^	Expressys
pZS21MCS	*E. coli*—*K. pneumoniae* shuttle vector, P_LtetO-1_, pSC101 ori, *Km* ^R^	Expressys
pZA31MCS::*ptsG*	pZA31MCS derivative containing *ptsG*, P_LtetO-1_, p15A ori, Cm^R^	This study
pZS21MCS::*scrAB*	pZS21MCS derivative containing *scrAB*, P_LtetO-1_, pSC101 ori, *Km* ^R^	This study

**Fig. 2:**
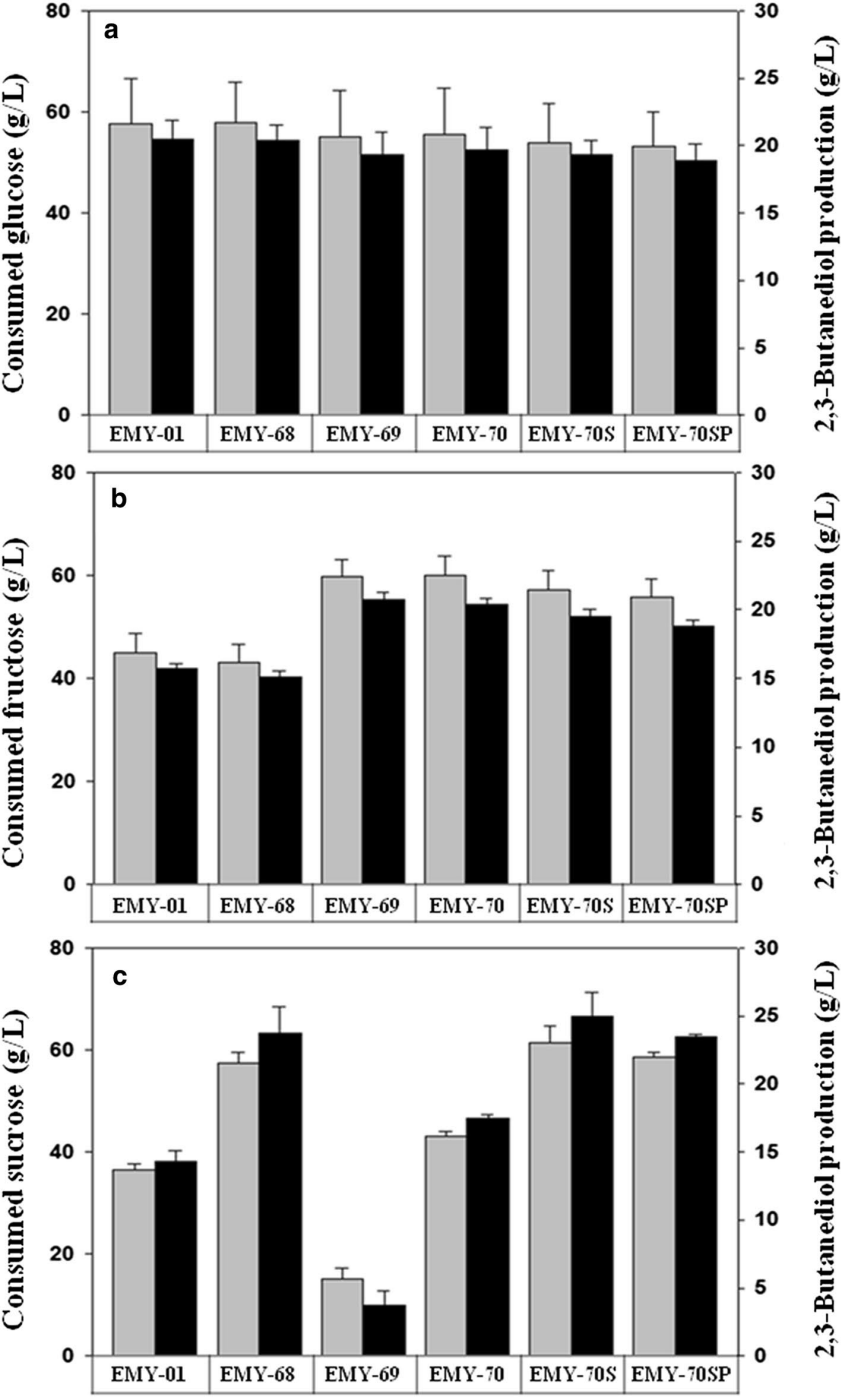
Comparison of consumed concentration of **a** glucose, **b** fructose, and **c** sucrose by *E. aerogenes* mutants in 10 h of flask cultivation. *Gray bars* concentration of consumed sugars; *black bars* 2,3-butanediol production. *Error bars* represent the standard deviations of three experiments.

Flask cultivations of EMY-01, EMY-68, EMY-69, and EMY-70 were conducted with 80 g/L of three different carbon sources (fructose, glucose, and sucrose) for 10 h to confirm the effect of *cra* deletion on sugar utilization (Fig. 2; Additional file 2). The removal of *scrR,* a transcriptional repressor of the *scr* regulon for sucrose catabolism, caused considerable enhancement of sucrose utilization, but did not affect the utilization of fructose and glucose, in accordance with a previous report [19]. The disruption of *cra* did not affect the consumption of glucose, while significantly increasing fructose utilization by 32.9 and 39.0% in EMY-69 and EMY-70, respectively (Fig. 2a, b). The improvement in fructose utilization increased 2,3-butanediol production by 32.5 and 35.1%, respectively. Interestingly, the removal of *cra* repressed the utilization of sucrose significantly. The sucrose consumption of EMY-69 and EMY-70 was reduced by 58.2 and 24.6%, respectively, compared to that of their parent strains (Fig. 2c).

**Fig. 3:**
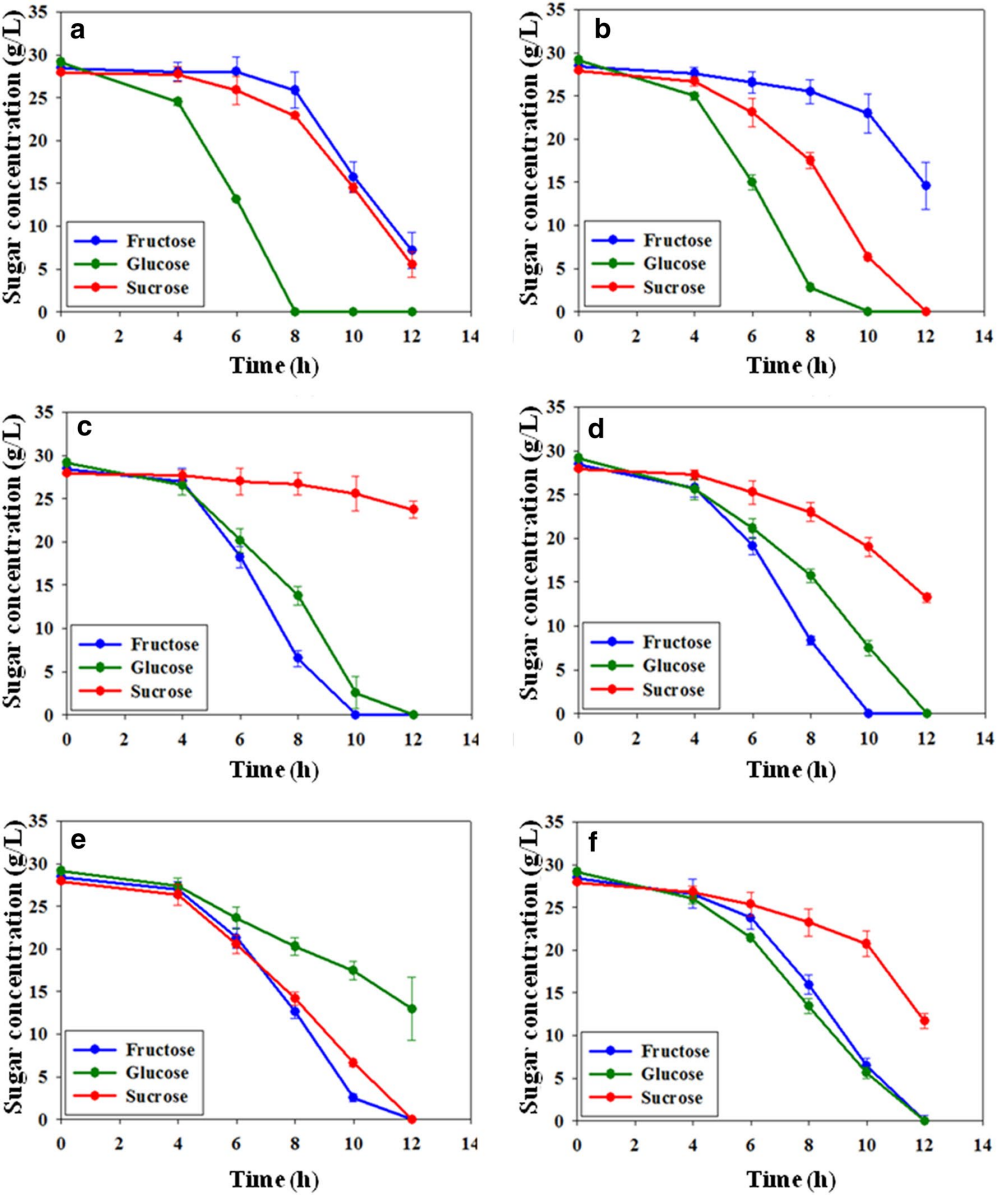
Comparison of flask cultivations of **a** EMY-01, **b** EMY-68, **c** EMY-69, **d** EMY-70, **e** EMY-70S, and **f** EMY-70SP with the consumption of sugars. *Error bars* represent the standard deviations of three experiments.

The effect of *cra* mutation was also confirmed in flask cultivation with a mixture of the three sugars. As shown in Fig. 3a, b, glucose was the preferred carbon source of EMY-01 and EMY-68. After glucose was depleted, EMY-01 utilized fructose and sucrose at similar rates, while EMY-68 consumed sucrose faster than fructose due to the disruption of *scrR.* Meanwhile, the most preferred sugar of the *cra*-deficient mutants was fructose. The fructose consumption rates of EMY-69 and EMY-70 increased by 124.0 and 420.5%, respectively, compared to that of their parent strains in 10 h of flask cultivation with mixed sugars. Furthermore, a significant decrease (81.3%) in the sucrose consumption of EMY-69 was also observed with 12 h of flask cultivation with mixed carbon sources, resulting in a 24.1% reduction of 2,3-butanediol production (Fig. 3; Additional file 3).

Transcription of the fructose operon is repressed when Cra is bound to the operator, which is located on the downstream region of the RNA polymerase binding site [24]. Whereas, derepression of the fructose operon takes place when cytoplasmic glycolytic catabolites, such as fructose-1-phosphate and fructose-1,6-bisphosphate, bind to the Cra protein, causing it to dissociate from the operator. Our results demonstrate that *cra* deletion improves the consumption rate of fructose significantly and removes CCR with respect to fructose utilization, which increased 2,3-butanediol production. At the same time, a significant decrease in the sucrose uptake rate was observed with deletion of Cra, indicating a significant interaction between the *scr* regulon and Cra. The efficient utilization of sucrose is essential for maximizing the use of sugarcane molasses, because 70% of the carbon contained in sugarcane molasses is sucrose. However, the regulation of sucrose catabolism by Cra remains unexplored in enteric bacteria.

### Regulation of genes involved in sucrose catabolism in *cra*-deficient *E. aerogenes*

**Fig. 4:**
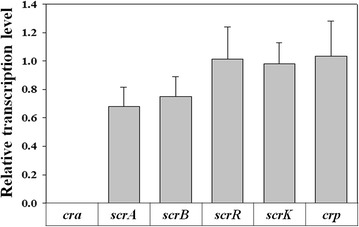
Real-time PCR results of genes involved in sucrose catabolism by the deletion of the *cra* gene*. The transcription level of genes in EMY*-*01 was used as the control. Error bars* represent the standard deviations of three experiments.

To observe the mechanism of Cra regulation of sucrose catabolism, reverse transcription and real-time PCR was conducted with a *cra*-deficient mutant, which was grown in sucrose medium. Six genes related to sucrose utilization (*scrA*, *scrB*, *scrR*, *scrK*, *cra*, and *crp*) were selected for the reverse transcription and real-time PCR assay. The transcription level of genes involved in the sucrose catabolism of EMY-01 and EMY-69 was compared in the mid-exponential growth phase (6 h), using *gapA* as a reference gene [28]. As shown in Fig. 4, there were no significant differences in the relative expression levels of *scrR*, *scrK*, or *crp* in the strains, whereas the transcription levels of *scrA* and *scrB* decreased by 32.0 and 24.9%, respectively, by disruption of the *cra* gene.

In *E. aerogenes*, the *scr* regulon for sucrose catabolism consists of four genes, which encode ATP-dependent fructokinase (ScrK), PTS EII transport protein (ScrA), sucrose-6-phosphate hydrolase (ScrB), and sucrose-dependent regulator (ScrR) [29, 30]. The *scr* regulon contains two independent promoters, one in front of the *scrK* and a second in front of the *scrAB* operon, both of which are negatively regulated by ScrR (Additional file 1) [31]. In *E. coli*, the sequence of the Cra-binding site (Cra box), TGAAACGTTTCA, has been well characterized [16]. Interestingly, a sequence similar to the consensus sequence of the Cra box, TAAAACGTTTTA, was found in the *scrAB* promoter region of *E. aerogenes*. In addition, no significant change in the transcription level of *crp* was observed in our results when *cra* was disrupted. These results demonstrate that Cra directly binds to the *scrAB* promoter region to regulate its expression.

The CCR mechanisms by Crp and Cra are complex and interconnected. Indeed, Cra regulates the utilization of several carbon sources, such as xylose, glycerol, lactose, and sorbitol, through activating expression of the *crp* gene, even though Cra does not regulate the transcription of relevant catabolic genes directly [13]. In this experiment, we demonstrated the sugar consumption genes can be modulated by *cra* deletion, and that regulation is independent with Crp. This result is another example of controlling CCR. Previously Ji et al. tried it by overexpression of *crp*(in) gene, which made *Klebsiella oxytoca* utilize glucose and xylose simultaneously and resulted in higher growth and 2,3-butanediol productivity [32].

### Effects of *scrAB* and *ptsG* overexpression on the sugar utilization preference in sugarcane molasses

The decreased sucrose utilization by deletion of *cra* in EMY-69 was partially relieved by *scrR* mutation, shown in EMY-70 (Fig. 2c). However, sucrose utilization in that strain was not as efficient as glucose or fructose. Therefore, the *scrAB* operon was cloned in the expression vector pZS21MCS and overexpressed in EMY-70 to increase sucrose utilization. The resulting strain, EMY-70S, showed considerably enhanced sucrose utilization, as shown in Fig. 2c. EMY-70S consumed 61.5 g/L sucrose for 10 h of flask culture using sucrose as a sole carbon source, representing an increase of 42.2% compared to that of EMY-70. In flask cultivation of EMY-70S with mixed sugars, the consumption rate of sucrose improved to a similar rate as that of fructose consumption (Fig. 3e). Interestingly, glucose was the carbon source with the lowest priority among the three carbon sources in EMY-70S. The consumption of glucose was reduced by 46.0% compared to that of its parent strain (Figs. 3d, e).

Thus, we attempted to resolve the reduced efficiency of glucose utilization in EMY-70S at the genetic level. In glucose catabolism, the EIIBC^Glc^ complex encoded by *ptsG* is known to be a major controller of glucose flux in *E. coli* [33, 34]. Therefore, a strain overexpressing the *ptsG* gene was constructed from EMY-70S and named EMY-70SP. Although the overexpression of *ptsG* did not affect the carbon source utilization of a sole carbohydrate, such as fructose, glucose, or sucrose, the efficiency of glucose utilization was restored in flask cultivations with a mixture of sugars (Figs. 2, 3f). In EMY-70SP, the consumption rate of sucrose was slightly reduced. Among the carbohydrate consumption pathways (Fig. 1), G6P is converted not only from glucose by the *ptsG* gene product, but also from sucrose by a series of enzymes involved in the *scrAB* operon. Therefore, the result suggested that overexpression of the *ptsG* gene presumably activated glucose transport with G6P formation, which resulted in the relative repression of G6P synthesis derived from sucrose.

As shown in the Additional file 3, there were no significant differences in the total concentration of the carbon source consumed by *E. aerogenes* mutants when the flask cultivations were performed with mixed sugars. These results indicate that the maximum carbon source consumption through glycolysis might be reached, but the sugar preference was modulated by genetic engineering. Still, predictable control of sugar preference would be very beneficial for efficient utilization of biomass that contains a variety of carbon sources. This is because the concentration and content ratio of sugars contained in biomass vary depending on the type of biomass resources and local climate and soil conditions even the same type of biomass [35–37].

### Fed-batch fermentation with *E. aerogenes* mutants using sugarcane molasses

**Fig. 5:**
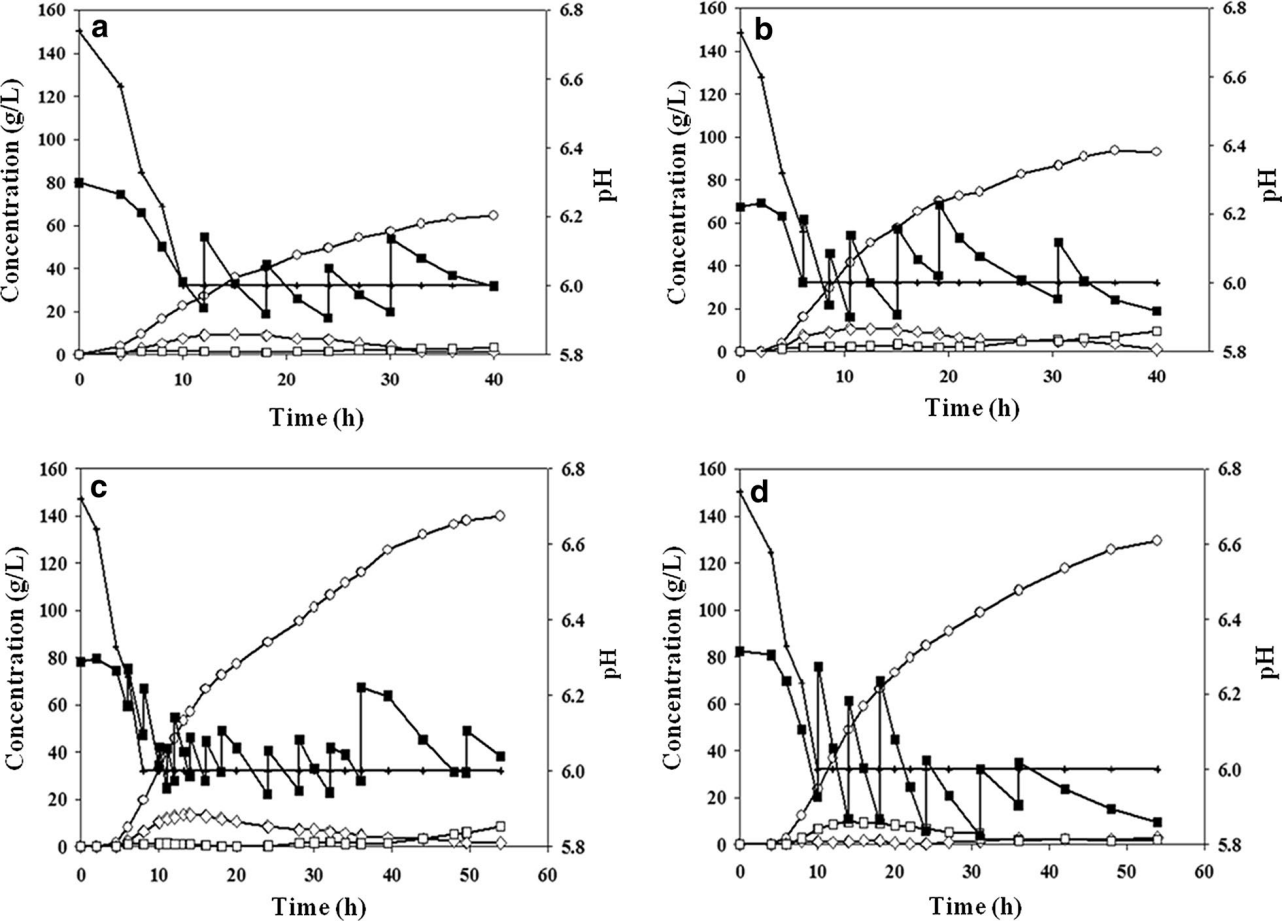
Results of fed-batch fermentations using sugarcane molasses with **a** EMY-01, **b** EMY-68, **c** EMY-70S and **d** EMY-70SP. *Symbols* represent 2,3-butanediol (*white circles*), total sugars (*closed squares*), pH (*pluses*), ethanol (*open diamonds*), and acetoin (*open squares*).

**Fig. 6:**
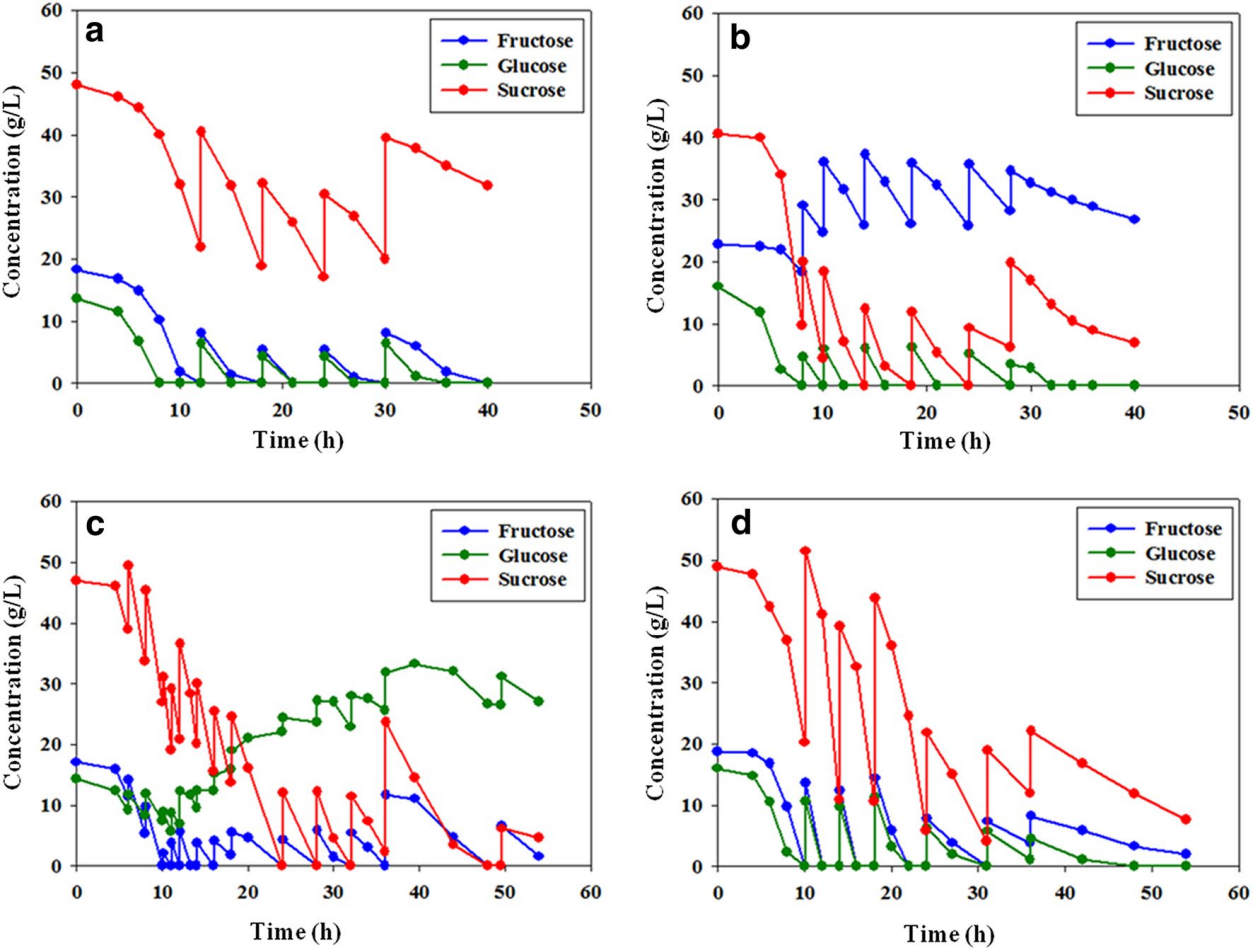
Comparison of fed-batch fermentations of **a** EMY-01, **b** EMY-68, **c** EMY-70S and **d** EMY-70SP with the consumption of sugars.

To improve 2,3-butanediol production from sugarcane molasses, a fed-batch fermentation was performed with EMY-70S. As shown in Fig. 5c and Additional file 4, 2,3-butanediol production reached 140.0 g/L at 54 h of cultivation. To the best of our knowledge, this was by far the highest titer of 2,3-butanediol with *E. aerogenes* achieved by genetic engineering, and was 18.6% higher than that with glucose as a carbon source [27]. A total of 361.7 g/L of sugars in sugarcane molasses was consumed. Among the major byproducts, ethanol production reached 13.7 g/L at 14 h of fed-batch fermentation, after which its concentration was gradually decreased to 1.6 g/L at 54 h, in accordance with a previous report [19]. The production of acetoin, which is a precursor of 2,3-butanediol, was under 2.0 g/L until 40 h of fermentation, but its titer steadily increased to 8.6 g/L at 54 h. The acetoin accumulation is due to the reduced activity of 2,3-butanediol dehydrogenase (BudC) by a high concentration of 2,3-butanediol in the fermentation broth. The production of 2,3-butanediol by EMY-70S was prolonged until 54 h of cultivation, which was 50% longer than the fed-batch with both EMY-01 and EMY-68 (Figs. 5, 6). Even at 36 h of fed-batch fermentation, 2,3-butanediol productivity (g/L/h) of EMY-70S increased by 23.8% compared to that of EMY-68. These results indicate that disruption of both *scrR* and *cra* genes presumably relieved the cell stress due to repeated catabolic shift according to the feeding of sugarcane molasses in fed-batch fermentation. However, there was significant accumulation (30 g/L) of glucose in the medium after the fermentation period (Fig. 6c).

To observe the effect of *ptsG* overexpression on glucose consumption, fed-batch fermentation was performed with EMY-70SP. As shown in Fig. 5d and Additional file 4, 335.58 g/L of sugars in sugarcane molasses was consumed, and 2,3-butanediol production reached 129.36 g/L at 54 h of cultivation. Although 2,3-butanediol production was decreased by 7.6% compared to that of EMY-70S, all three carbons in sugarcane molasses were used efficiently (Fig. 6d). The metabolic burden according to overexpression of the *scrAB* operon and *ptsG* gene may be the main reason for the slight reduction observed in fed-batch fermentation with EMY-70SP. These results demonstrate the advantage of genetic engineering in utilizing mixtures of sugars contained in biomass under the fermentation process [38].

## Conclusions

The efficient utilization of biomass is necessary in order to develop an economic biorefinery industry. In this study, sugarcane molasses was used as a cheap feedstock for 2,3-butanediol production with metabolically engineered *E. aerogenes*. For efficient utilization of sugarcane molasses, the removal of transcriptional repressors enabled the mutant strain to metabolize all sugars in sugarcane molasses simultaneously, which increased fermentation duration and 2,3-butanediol productivity. In addition, the carbon preference was modulated by overexpression of key genes involved in each carbon catabolism, which relieved the accumulation of certain carbon sources in fed-batch fermentation. The metabolic engineering approach provided much higher 2,3-butanediol production and efficient utilization of carbon sources involved in sugarcane molasses. The strategy developed in this study is a promising approach for the biorefinery industry, through employing sugar mixtures derived from inexpensive biomass.

## Methods

### Construction of the gene-deficient mutants

All *E. aerogenes* strains were derived from the wild-type strain KCTC 2190 (Korean Collection for Type Culture), for which the genome has been sequenced [39]. Previously, the lactate dehydrogenase (LdhA, accession number YP_004594301.1) and sucrose regulator (ScrR, accession number YP_004593287.1) were disrupted from the KCTC 2190 genome by using a *λ* red recombination method [40], generating EMY-01 (*△ldhA*) [27] and EMY-68 (*△ldhA△scrR*) [19], respectively. In this study, the gene encoding catabolite repressor/activator, Cra (previously designated *fruR,* accession number YP_004592435.1), was deleted from the genome of EMY-01 and EMY-68 in a similar manner, and the resulting strains were named EMY-69 (*△ldhA△cra*) and EMY-70 (*△ldhA△scrR△cra*), respectively. The *cra*_FKF_fw and *cra*_FKF_rv primers were used for *cra* disruption, which was confirmed by colony PCR with the primers *cra*_con_A and *cra*_con_B. All resulting strains, along with primers and plasmids, used in this study are listed in Tables 1 and 2.

**Table 2:** Primers used in this study

Primer name	Primer sequence (5′ → 3′)^a^
*cra*_FKF_fw	GTATGTCTATTTAATGGTTGTTTTTTGTACTTCTTACCCAAGGGGCAATTGTGTAGGCTGGAGCTGCTTC
*cra*_FKF_rv	CCATCTGGCGAATAACCTACGAAGAATCTTAACCTTTTTTCGCAAATGAACCTCCTTAGTTCCTATTCC
*cra*_con_A	ACGTAAAAACAGCCCGACAC
*cra*_con_B	CGCTTTTTCTTGCACCATTT
pZS21_*scrAB*_fw	TTT**AAGCTT**ATGGATTTTCAACAGATTTCTCG
pZS21_*scrAB*_rv	AAA**CCCGGG**GCCTGAAAGCAAAACGCTTA
pZA31_*ptsG*_fw	TTT**AAGCTT**ACTCAGGAGCACTCTCAATTATGTT
pZA31_*ptsG*_rv	AAA**GGATCC**TTAGCTATTGCGGATGTACTCA
RT_*scrA*_fw	TCGGCGGTAACCCTTATCTT
RT_*scrA*_rv	ATTAGCCATCGCCCAGATAG
RT_*scrB*_fw	TGCTACACCGGTAATGTGAAAT
RT_*scrB*_rv	TAGAATTCGAAGCCGCTATCC
RT_*scrR*_fw	CCGGCGTACAGCTGCTTAT
RT_*scrR*_rv	ACGTAGTACCCCTTCCAGCAA
RT_*scrK*_fw	TCAGCCATCTTTCTTTAGATCC
RT_*scrK*_rv	CGGGAAGTGAATATGCTGTTG
RT_*cra*_fw	CCGTATTGCGAACTATCTGGA
RT_*cra*_rv	TAAACAGCAGTTGCGGCATT
RT_*crp*_fw	ATCAAAGAGCACGCTGATTC
RT_*crp*_rv	CCAGCATCTTCAGAATACGG
RT_*gapA*_fw	TTGGTGTTGACGTTGTTGCT
RT_*gapA*_rv	TTCGTAGGACGCTGCTTTTT

^a^Underlined sequences are the homologous sequences with the target genes of *E. aerogenes*. Restriction sites highlighted in bold.

### Construction of plasmids

*Enterobacter aerogenes* KCTC 2190 was cultured in Luria–Bertani medium overnight, and then total genomic DNA was extracted using the Wizard Genomic DNA Purification Kit (Promega, WI, USA). For *scrAB* overexpression, the *scrAB* fragment (a 2,771-bp segment of truncated *scrAB* gene, accession number YP_004593289.1 and YP_004593288.1) was amplified by PCR using the genomic DNA as a template and the primers pZS21*_scrAB_*fw and _rv at an annealing temperature (Tm) of 62.4°C. The PCR mixture consisted of 100 ng of genomic DNA, 200 μmol of dNTPs, 0.5 pmol each primer, 10 μL of 5× Phusion GC buffer, 1.5 μL of DMSO, and 1.0 unit of Phusion DNA polymerase (NEB, MA, USA) in a total volume of 50 μL. The amplified DNA fragments were cloned into the pZS21MCS plasmid joined by the *Hind*III and *Xma*I restriction sites using the DNA Ligation Kit Mighty Mix (TaKaRa Bio Inc., Shiga, Japan). The *E. coli* strain DH5α was used for amplification and confirmation of the constructed plasmid. The resulting plasmid was verified by sequencing. The *ptsG* fragment (a 1,434-bp segment of truncated *ptsG* gene, accession number YP_004593473.1) was amplified by PCR using the corresponding primers. The PCR mixture composition and procedure were the same as that of *scrAB*, but the annealing temperature was 64°C. The amplified *ptsG* fragments were cloned into the pZA31MCS plasmid after digestion with *Hind*III and *Bam*HI. The plasmids and primers used in this study are listed in Tables 1 and 2.

### Media and cultivation conditions

The fermentation medium to produce 2,3-butanediol contained (per L): 3 g KH_2_PO_4_, 6.8 g Na_2_HPO_4_, 0.75 g KCl, 5.35 g (NH_4_)_2_SO_4_, 0.28 g Na_2_SO_4_, 0.26 g MgSO_4_·7H_2_O, 0.42 g citric acid, 5 g yeast extract, 10 g casamino acid, and 0.3 mL microelement solution (1 L) containing 34.2 g ZnCl_2_, 2.7 g FeCl_3_·6H_2_O, 10 g MnCl_2_·4H_2_O, 0.85 g CuCl_2_·2H_2_O, and 0.31 g H_3_BO_3_, as described previously [19]. In a flask culture, 80 g/L of the individual sugars (glucose, fructose, or sucrose) was added to the medium as the sole carbon source, and the total 90 g/L mixture of sugars, containing 30 g/L each of glucose, fructose, and sucrose, was added to the medium for the sugar mixture cultivation. For pH neutralization of the flask cultivation, 5% calcium carbonate (CaCO_3_) was added to the medium before cultivation. The flask, sealed with a silicon stopper, was incubated at 250 rpm and 37°C in a 250-mL flask containing 50 mL medium with appropriate antibiotics or inducers, when needed, at the following concentrations: kanamycin (50 μg/mL), chloramphenicol (50 μg/mL), and anhydrotetracycline (50 ng/mL). The fed-batch fermentation was carried out in a 5-L stirring bioreactor (Bio Control and System, Daejeon, Korea) with a working volume of 3 L. The seed culture prepared previously was inoculated (5%, v/v) into the fermentation medium with an initial pH of 6.8. The pH value of the fermentation medium was decreased gradually to 6.0, and then maintained by the automatic addition of 5 M NaOH. During the fed-batch fermentation process, the operating temperature, airflow, and agitation speed were maintained at 37°C, 1.5 vvm, and 280 rpm, respectively. Antifoam 204 (Sigma, MO, USA) was added when needed. Brazilian sugarcane molasses was used, and its sugar content was 87 g/L fructose, 81 g/L glucose, and 387 g/L sucrose, as described previously [19]. The sterilized sugarcane molasses was fed before the sugar was depleted, and the sugar concentration was maintained under 80 g/L during fed-batch fermentation.

### Quantification of the transcription level of genes involved in sucrose catabolism by real-time PCR

Total RNA isolation was performed as previously described [41]. Briefly, cells (1 × 10^9^) were harvested at the mid-exponential phase (6 h) and, then, the total RNA in the cell was stabilized using the bacterial reagent RNAprotect (Qiagen, Hilden, Germany). Cell lysis was conducted with TE buffer containing lysozyme (400 μg/mL), and total RNA isolation was carried out using an RNeasy Mini Kit (Qiagen) following the manufacturer’s protocol. To synthesize the cDNA from total RNA, SuperScript^®^ Reverse Transcriptase (Invitrogen, CA, USA) was used. Real-time PCR was performed using the synthesized cDNA as a template (Model: MJ Mini thermocycler, Software: Opticon Monitor 3, Bio-Rad, CA, USA) with six sets of PCR primers and a SYBR Green mix (Prime Q-Master mix, Genet Bio, Daejon, Korea). The primers used in real-time PCR are listed in Table 2. The transcription levels of the five genes involved in sucrose catabolism were normalized to the housekeeping gene glyceraldehyde 3-phosphate dehydrogenase (*gapA*). The experiment was repeated three times independently.

### Analysis methods

Cell density was monitored by measurement of the optical density at 600 nm (OD_600_) with a UV/Visible spectrophotometer (DU730, Beckman Coulter, CA, USA). The concentrations of metabolites, obtained from cultivation, were measured by high-performance liquid chromatography (Waters HPLC 1500 series, MA, USA), equipped with a refractive index (RI) detector at 45°C. The amounts of fructose, glucose, and sucrose were measured using a High Performance Carbohydrate Column (Waters) at 35°C, and 80% acetonitrile was used as the mobile phase. Organic acids, 2,3-butanediol, acetoin, and ethanol, were measured using a Sugar SH1011 column (Shodex, Tokyo, Japan) at 75°C, and 10 mM sulfuric acid was used as the mobile phase. The flow rate of both mobile phases was maintained at 0.5 mL/min.

## Additional files

Additional file 1:
**Figure S1.** Regulation mechanism related to the utilization of sugars involved in sugarcane molasses and the strategies for constructing the 2,3-butanediol-producing strain in this study. Symbols represent deleted genes (red cross) and overexpressed genes (blue box). Additional file 2:
**Table S1.** Comparison of metabolite profiles obtained from *E. aerogenes* mutants using sole carbon sources in 10 h of flask cultivation. Additional file 3:
**Table S2.** Comparison of metabolite profiles obtained from *E. aerogenes* mutants using mixed carbon sources in 12 h of flask cultivation. Additional file 4:
**Table S3.** Comparison of fed-batch fermentation with EMY-01, EMY-68, EMY-70S, and EMY-70SP using sugarcane molasses.

## Abbreviations

Cra: catabolite repressor/activator
CCR: carbon catabolite repression
PTS: phosphotransferase system
G6P: glucose-6-phosphate
F6P: fructose-6-phosphate
F1,6BP: fructose-1,6-bisphosphate
PCR: polymerase chain reaction
Ldh: lactate dehydrogenase
OD: optical density
ScrA: EII transport protein for sucrose
ScrB: sucrose-6-phosphate hydrolase
ScrK: fructokinase
ScrR: transcriptional repressor of *scr* regulon
PtsG: EIIBC^Glc^ complex
FruA: EIIBC^fru^ complex
FruB: EIIA^fru^ component
FruK: fructose-1-phosphate kinase
Crp: cyclic AMP receptor protein
