# Supplementary material for: Alleviation of carbon catabolite repression in Enterobacter aerogenes for efficient utilization of sugarcane molasses for 2,3-butanediol production
Source: Biotechnol Biofuels. 2015 Jul 31;8:106. doi: 10.1186/s13068-015-0290-3 (PMC4521459; doi:10.1186/s13068-015-0290-3)
Supplement: Additional file 1: — Figure S1. Regulation mechanism related to the utilization of sugars involved in sugarcane molasses and the strategies for constructing the 2,3-butanediol-producing strain in this study. Symbols represent deleted genes (red cross) and overexpressed genes (blue box). [file 13068_2015_290_MOESM1_ESM.docx]

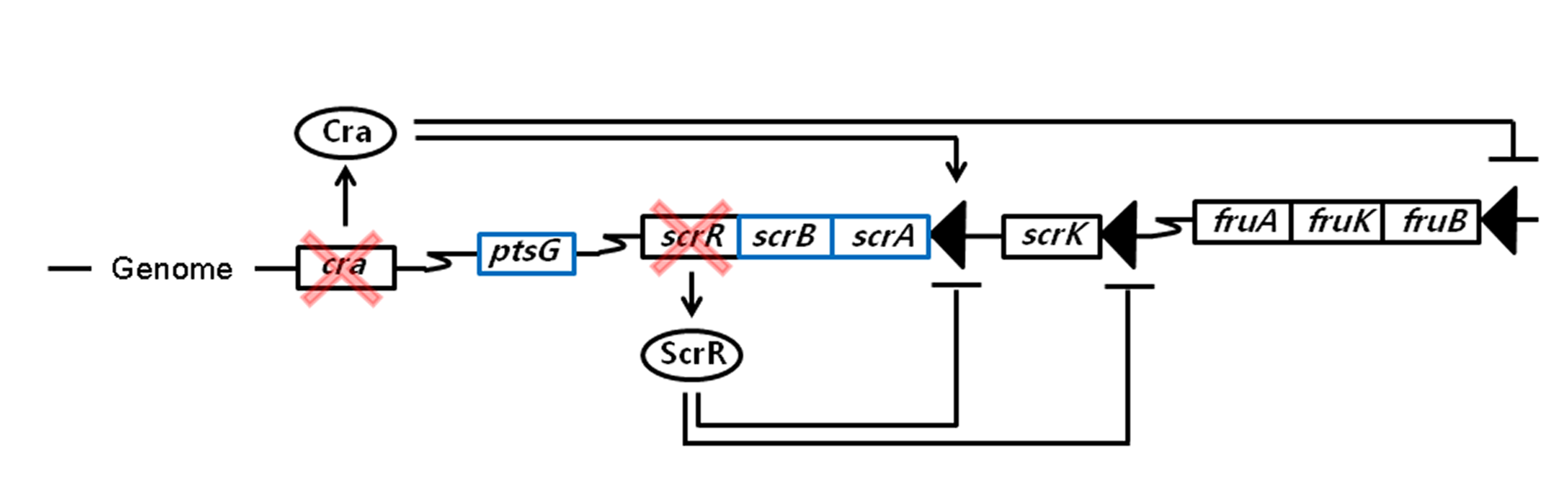


**Figure S1. Proposed regulation mechanism related to the utilization of sugars in sugarcane molasses and the strategies for constructing the 2,3-butanediol producing strain in this study.**

Symbols represent deleted genes (red cross) and over-expressed genes (blue box)
