# Supplementary material for: Alleviation of carbon catabolite repression in Enterobacter aerogenes for efficient utilization of sugarcane molasses for 2,3-butanediol production
Source: Biotechnol Biofuels. 2015 Jul 31;8:106. doi: 10.1186/s13068-015-0290-3 (PMC4521459; doi:10.1186/s13068-015-0290-3)
Supplement: Additional file 2: — Table S1. Comparison of metabolite profiles obtained from E. aerogenes mutants using sole carbon sources in 10 h of flask cultivation. [file 13068_2015_290_MOESM2_ESM.docx]

## Additional file 2 - Comparison of metabolite profiles obtained from *E. aerogenes* mutants using sole carbon sources in 10 h of flask cultivation^a^

| **Carbon source** | **Glucose** | | | | | |  | **Fructose** | | | | | |  | **Sucrose** | | | | | |  |
| --- | --- | --- | --- | --- | --- | --- | --- | --- | --- | --- | --- | --- | --- | --- | --- | --- | --- | --- | --- | --- | --- |
| **Strain** | **EMY-**  **01** | **EMY-68** | **EMY-69** | **EMY-70** | **EMY-70S** | **EMY-70SP** |  | **EMY-01** | **EMY-68** | **EMY-69** | **EMY-70** | **EMY-70S** | **EMY-70SP** |  | **EMY-01** | **EMY-68** | **EMY-69** | **EMY-70** | **EMY-70S** | **EMY-70SP** |  |
| **Initial carbon source (g/L)** | **80.2** | | | | | |  | **83.7** | | | | | |  | **74.7** | | | | | |  |
| **Consumption of carbon source (g/L)** | **57.8** | **57.8** | **55.0** | **55.6** | **54.0** | **53.3** |  | **45.0** | **43.1** | **59.8** | **59.9** | **57.2** | **55.8** |  | **36.4** | **57.4** | **15.2** | **43.2** | **61.5** | **58.7** |  |
| **2,3-Butanediol production**  **(g/L)** | **20.5** | **20.4** | **19.3** | **19.7** | **19.3** | **18.9** |  | **15.7** | **15.1** | **20.8** | **20.4** | **19.5** | **18.9** |  | **14.3** | **23.7** | **3.8** | **17.5** | **24.9** | **23.5** |  |
| **Acetoin**  **production (g/L)** | **0.8** | **0.8** | **0.9** | **0.9** | **1.0** | **0.9** |  | **0.8** | **0.8** | **1.0** | **0.5** | **1.0** | **0.9** |  | **0.7** | **0.8** | **0.5** | **1.0** | **0.4** | **0.4** |  |
| **Ethanol**  **production (g/L)** | **10.3** | **9.9** | **9.9** | **10.0** | **9.6** | **9.1** |  | **7.4** | **7.2** | **10.1** | **10.6** | **9.9** | **9.7** |  | **7.8** | **12.0** | **3.5** | **9.6** | **13.6** | **13.5** |  |
| **Lactate**  **production (g/L)** | **0.4** | **0.4** | **0.2** | **0.2** | **0.2** | **0.2** |  | **0.1** | **ND** | **ND** | **ND** | **ND** | **ND** |  | **ND** | **0.1** | **ND** | **ND** | **ND** | **ND** |  |
| **Succinate production (g/L)** | **2.2** | **2.2** | **2.3** | **2.4** | **2.4** | **2.7** |  | **3.1** | **3.0** | **2.5** | **2.4** | **2.3** | **2.1** |  | **3.9** | **3.0** | **2.7** | **3.2** | **3.3** | **3.5** |  |
| **Initial pH** | **6.73** | | | | | |  | **6.85** | | | | | |  | **6.86** | | | | | |  |
| **Final pH** | **5.60** | **5.60** | **5.62** | **5.61** | **5.62** | **5.58** |  | **5.65** | **5.67** | **5.60** | **5.61** | **5.62** | **5.57** |  | **5.54** | **5.43** | **5.62** | **5.56** | **5.36** | **5.34** |  |

*^a^* The experiment was repeated three times independently.
