# Supplementary material for: Alleviation of carbon catabolite repression in Enterobacter aerogenes for efficient utilization of sugarcane molasses for 2,3-butanediol production
Source: Biotechnol Biofuels. 2015 Jul 31;8:106. doi: 10.1186/s13068-015-0290-3 (PMC4521459; doi:10.1186/s13068-015-0290-3)
Supplement: Additional file 3: — Table S2. Comparison of metabolite profiles obtained from E. aerogenes mutants using mixed carbon sources in 12 h of flask cultivation. [file 13068_2015_290_MOESM3_ESM.docx]

**Additional file 3 – Comparison of metabolite profiles obtained from *E. aerogenes* mutants using mixed carbon sources in 12 h of flask cultivation^a^**

|  | | **EMY-01** | **EMY-68** | **EMY-69** | **EMY-70** | **EMY-70S** | **EMY-70SP** |
| --- | --- | --- | --- | --- | --- | --- | --- |
| **Intial sugar** | **Fructose** | **28.45** | | | | | |
|  | **Glucose** | **29.16** | | | | | |
|  | **Sucrose** | **27.94** | | | | | |
|  | **Total** | **85.56** | | | | | |
| **Consumed sugar** | **Fructose** | **21.29** | **13.85** | **28.45** | **28.45** | **28.45** | **28.45** |
|  | **Glucose** | **29.16** | **29.16** | **29.16** | **29.16** | **16.19** | **29.16** |
|  | **Sucrose** | **22.41** | **27.94** | **4.21** | **14.71** | **27.94** | **13.24** |
|  | **Total** | **78.87** | **70.95** | **61.82** | **72.32** | **72.58** | **72.85** |
| **2,3-BDO production (g/L)** | | **30.25** | **28.65** | **22.95** | **27.10** | **28.60** | **28.36** |
| **Acetoin production (g/L)** | | **0.97** | **2.09** | **1.28** | **1.18** | **1.08** | **1.14** |
| **Ethanol production (g/L)** | | **14.93** | **14.20** | **12.17** | **14.23** | **14.79** | **14.66** |
| **Succinate production (g/L)** | | **3.87** | **3.71** | **3.44** | **4.08** | **4.10** | **4.70** |
| **Acetate production (g/L)** | | **ND** | **0.34** | **1.34** | **1.15** | **0.77** | **0.31** |
| **Initial pH** | | **6.74** | | | | | |
| **Final pH** | | **5.33** | **5.38** | **5.38** | **5.25** | **5.26** | **5.25** |

*^a^* The experiment was repeated three times independently.
