# Supplementary material for: Alleviation of carbon catabolite repression in Enterobacter aerogenes for efficient utilization of sugarcane molasses for 2,3-butanediol production
Source: Biotechnol Biofuels. 2015 Jul 31;8:106. doi: 10.1186/s13068-015-0290-3 (PMC4521459; doi:10.1186/s13068-015-0290-3)
Supplement: Additional file 4: — Table S3. Comparison of fed-batch fermentation with EMY-01, EMY-68, EMY-70S, and EMY-70SP using sugarcane molasses. [file 13068_2015_290_MOESM4_ESM.docx]

**Additional file 4 – Comparison of fed-batch fermentation with EMY-01, EMY-68, EMY-70S and EMY-70SP using sugarcane molasses**

|  | **EMY-01** | **EMY-68** | **EMY-70S** | **EMY-70SP** |
| --- | --- | --- | --- | --- |
| **Fermentation time (h)** | **36** | **36** | **54** | **54** |
| **Consumption of total sugars (g/l)** | **175.34** | **269.85** | **361.71** | **335.58** |
| **2,3-Butanediol production (g/l)** | **63.33** | **98.69** | **140.00** | **129.36** |
| **2,3-Butanediol yield (g/g)** | **0.361** | **0.366** | **0.387** | **0.385** |
| **Maximum 2,3-butanediol productivity (g/l/h)^a^** | **2.48** | **3.94** | **4.94** | **5.03** |
| **Acetoin production (g/L)** | **2.68** | **7.03** | **8.60** | **2.90** |
| **Ethanol production (g/L)** | **1.56** | **3.51** | **1.55** | **1.94** |

^a^ Maximum 2,3-butanediol productivity was calculated in the exponential phase (6 to 20 hours) that shows a linear pattern of 2,3-butanediol production.
